# Protective Effects of a Red Grape Juice Extract against Bisphenol A-Induced Toxicity in Human Umbilical Vein Endothelial Cells

**DOI:** 10.3390/toxics11040391

**Published:** 2023-04-21

**Authors:** Caterina Russo, Alessandro Maugeri, Ambrogina Albergamo, Giacomo Dugo, Michele Navarra, Santa Cirmi

**Affiliations:** 1 Department of Chemical, Biological, Pharmaceutical and Environmental Sciences, University of Messina, 98166 Messina, Italy; carusso@unime.it (C.R.); scirmi@unime.it (S.C.); 2 Department of Veterinary Sciences, University of Messina, 98168 Messina, Italy; amaugeri@unime.it; 3 Department of Biomedical, Dental, Morphological and Functional Images Sciences, University of Messina, 98100 Messina, Italy; aalbergamo@unime.it (A.A.); dugog@unime.it (G.D.); 4 Science4Life s.r.l., a Spin-Off of the University of Messina, 98168 Messina, Italy

**Keywords:** bisphenol A, oxidative stress, red grape juice extract, HUVECs, endothelial dysfunction, atherosclerosis, cytotoxicity

## Abstract

Human exposure to bisphenol A (BPA) occurs through the ingestion of contaminated food and water, thus leading to endothelial dysfunction, the first signal of atherosclerosis. *Vitis vinifera* L. (grape) juice is well known for its health-promoting properties, due to its numerous bioactive compounds among which are polyphenols. The aim of this study was to evaluate the protective effect of a red grape juice extract (RGJe) against the endothelial damage induced by BPA in human umbilical vein endothelial cells (HUVECs) as an in vitro model of endothelial dysfunction. Our results showed that RGJe treatment counteracted BPA-induced cell death and apoptosis in HUVECs, blocking caspase 3 and modulating p53, Bax, and Bcl-2. Moreover, RGJe demonstrated antioxidant properties in abiotic tests and in vitro, where it reduced BPA-induced reactive oxygen species as well as restored mitochondrial membrane potential, DNA integrity, and nitric oxide levels. Furthermore, RGJe reduced the increase of chemokines (IL-8, IL-1β, and MCP-1) and adhesion molecules (VCAM-1, ICAM-1, and E-selectin), caused by BPA exposure, involved in the primary phase of atheromatous plaque formation. Overall, our results suggest that RGJe prevents BPA-induced vascular damage modulating specific intracellular mechanisms, along with protecting cells, owing to its antioxidant capability.

## 1. Introduction

Bisphenol A (BPA), 2,2-Bis(4-hydroxyphenyl) propane, a common component of polycarbonate plastics and epoxy resins, is one of the major chemicals produced by humans in the world. Due to BPA leaching from these polymers under heat and acidic or basic conditions, humans are exposed to this pollutant mainly via contaminated foods but also beverages, dental sealants, baby bottles, food cans, and containers [1]. Owing to its extensive use and large presence in the environment, exposure to BPA is common among general populations worldwide. 

Several studies suggest that BPA has endocrine-disrupting properties and that it can be implied in the pathogenesis of a variety of human diseases mostly affecting reproductive systems [2,3], similar to other environmental pollutants [4], as well as cardiovascular systems [5]. In this frame, epidemiological studies reported that there is an association between exposure to BPA and an increased prevalence of cardiovascular diseases (CVDs), including atherosclerosis [6], coronary and peripheral artery disease [7,8], myocardial infarction [9], and angina pectoris [10], as well as their risk factors, such as hypertension [11] and diabetes [12]. At the basis of the pathogenesis of these illnesses, a pivotal role is played by oxidative stress, endothelial activation, and dysfunction, which the latter represents the early step in the development and progression of atherosclerosis [13]. 

Atherosclerosis is an inflammatory vessel disorder characterized by plaque formation because of monocyte-derived macrophages that ultimately develop into lipid-laden foam cells [14]. In the initial stages of atherosclerotic plaque expansion, circulating monocytes are recruited by activated endothelial cells followed by monocyte adhesion and successive transmigration into the intima [14]. These processes are driven by pro-inflammatory chemokines, such as interleukin-8 (IL-8) and monocyte chemoattractant protein-1 (MCP-1), as well as adhesion molecules, such as intracellular adhesion molecule-1 (ICAM-1) and vascular cell adhesion molecule-1 (VCAM-1) [15].

An important mechanism for vascular endothelial dysfunction in atherosclerosis is the high levels of reactive oxygen species (ROS) [16]. This is because increased vascular ROS generation, including superoxide (O_2_^•−^) and hydrogen peroxide (H_2_O_2_), is acknowledged to cause oxidative stress, mitochondrial damage, reduction of vasoprotective nitric oxide (NO), and activation of pro-inflammatory signaling cascades [16]. Moreover, excessive ROS production causes the oxidation of macromolecules inducing cell apoptosis [16]. 

Epidemiological studies demonstrated that the high intake of polyphenols-rich food is related to the low risk of various CVDs [17,18]. Grapes are one of the main natural dietary sources of polyphenols. These bioactive molecules are associated with health-promoting properties such as antioxidant, anti-atherogenic, antithrombotic, antimicrobial, anti-inflammatory activities, and protective effects against non-communicable diseases, including cancer and cardiovascular, metabolic, and neurodegenerative disorders [19,20].

On these bases, the aim of this study was to evaluate the protective effects of red grape juice extract (RGJe), from *Vitis vinifera* L. (Vitaceae), against the endothelial damage induced by BPA in human umbilical vein endothelial cells (HUVECs) as an in vitro model of endothelial dysfunction.

## 2. Materials and Methods

### 2.1. Drug

RGJe was provided by the company “Bono & Ditta” (Campobello di Mazara, Trapani, Italy). The extract in liquid form has been produced by passing the must-mute through columns equipped with adsorbent resins which retain polyphenolic compounds. Then, molecules were eluted with 4% NaOH and immediately passed through cationic resins, which allows to change the biomolecules in acid form. The product was collected, filtered, and stored at +4 °C for a short time. Finally, liquid extract (500 mL) was transformed into a dry powder (12.452 g) by lyophilization, and small aliquots were stored at −20 °C until use. At the beginning of each experiment, an aliquot of RGJe was defrosted and dissolved in RPMI cell culture medium at the desired concentrations.

### 2.2. Chemical Characterization of RGJe

The polyphenol profile of RGJe was investigated by reversed-phase ultrahigh-performance liquid chromatography coupled with mass spectrometry (RP-UHPLC-MS). A sample aliquot (50 mg) was suspended in 5 mL of water/acetonitrile (50:50, *v*/*v*), filtered through a 0.2 µm syringe PTFE filter, and directly injected into a Shimadzu Prominence UFLC XR system (Shimadzu, Kyoto, Japan). The latter consists of a CBM-20A controller, an LC-20AD-XR binary pump system, a DGU-20A3R degasser, a CTO-20AC column oven, and a SIL-20A XR autosampler and is interfaced through an electrospray ionization (ESI) source to an LCMS-8040 triple quadrupole mass spectrometer (Shimadzu, Kyoto, Japan), as reported elsewhere [21]. Chromatographic separations occurred on a UPLC^®^ BEH C18 analytical column (2.1 × 100 mm, 1.7 µm, Waters, Milford, MA, USA) protected by a guard column with the same stationary phase Acquity UPLC^®^ BEH C18 VanGuard column (2.1 × 5 mm, particle size 1.7 µm, Waters). The mobile phase was composed of water (phase A) and acetonitrile acidified with 0.1% formic acid (phase B), and the optimized gradient profile was as follows: 0–5 min, 5% B; 5–12 min 5–25% B; 12–15 min, 25–45% B; and 15–20 min, 75% B; this also included final column washing and re-equilibrating steps. Oven temperature and injection volume were set at 35 °C and 3 μL, respectively. The flow rate was 0.5 mL/min, and a stainless-steel splitting device (VICI AG International, Schenkon, Switzerland) assured that only 300 µL/min of the flow was directed from the LC system to the ESI interface. The ionization source was used in negative ionization mode according to the following parameters: capillary voltage, 4.5 kV; heat block temperature, 400 °C; desolvation line (DL) temperature and voltage, 250 °C and −35 V; drying gas flow (N2), 15 L/min; and nebulizing gas flow (N2), 3 L/min. Spectra acquisition occurred in full scan mode over an *m*/*z* range of 100–800. 

The investigated compounds belonged to diverse polyphenol classes, and they were selected depending on standard availability and their occurrence in similar products [22,23]. For quantification purposes, an external calibration procedure was carried out by means of six-point calibration curves showing satisfactory linearity (r2 from 0.9986 (kaempferol-3-glucoronide) to 0.9996 (luteolin)). Triplicate measurements along with analytical blanks (i.e., acetonitrile) were acquired and elaborated by the LCMS solution Ver. 3.30 software (Shimadzu, Kyoto, Japan).

### 2.3. Evaluation of RGJe Antioxidant Activity through Abiotic Assays

The total phenolic content of RGJe was evaluated through the Folin–Ciocalteu [24] assay, while its total antioxidant activity was through the oxygen radical absorbance capacity (ORAC) assay, as previously reported [25]. The 2,2-Diphenyl-1-picrylhydrazyl (DPPH^•^) was employed to test the radical scavenging activity of our extract, in accordance with Lombardo and co-workers [26]. The reducing power of RGJe was determined, according to Ferlazzo and collaborators, through the potassium ferricyanide reducing power assay [27]. All tests were repeated three times, and the results are expressed as mean ± SEM. As standards, we employed gallic acid, Trolox, and ascorbic acid in relation to the assay performed.

### 2.4. Cell Culture

This study was conducted using the human umbilical vein endothelial cells (HUVECs) originally from American Type Culture Collection (ATCC; Rockville, MD, USA). HUVECs were cultured in uncoated polystyrene dishes in endothelial basal medium (EBM^TM^-2; Lonza, Walkersville, MD, USA) supplemented with Endothelial Cell Growth Medium-2 (EGM^TM^-2) BulletKit™ (Lonza) at 37 °C in a 5% CO_2_ air humified atmosphere and used between the third and sixth passages.

### 2.5. Cell Viability Assays

Cell viability was evaluated by the 3-(4,5-dimethylthiazole-2-yl)-2,5-diphenyl-tetrazolium bromide (MTT) test [28]. HUVECs were seeded into 96-well plates at a density of 1 × 10^4^ cells/well in a complete medium and left to attach for 24 h. Before evaluating the potential protective effect of RGJe against toxicity induced by BPA, we evaluated the effect of BPA treatment (ranging from 0 to 1000 µM) for 24 h and RGJe (ranging from 0 to 200 µg/mL) for 48 h in HUVECs, tracing the timings of the following experiments.

Then, cells were treated with RGJe at various concentrations (from 1 to 50 µg/mL) for 24 h and BPA 300 µM for an additional 24 h. Then, the plates were centrifuged at 1200 rpm for 10 min, supernatants were removed, and fresh media without phenol red containing 0.5 mg/mL of MTT (Sigma-Aldrich, Milan, Italy) was added to each well. Plates were put in the incubator for an additional 4 h. Afterwards, insoluble formazan crystals were dissolved in 100 µL HCl/isopropanol 0.1 N lysis solution. The absorbance was spectrophotometrically quantified through iMark™ microplate reader (Bio-Rad Laboratories, Milan, Italy) at a wavelength of 570 nm with reference at 630 nm. The viability was determined as the percentage of viable cells in treated cultures compared to those in untreated ones.

The protective effect of RGJe against BPA-induced cell death was also evaluated by bromo-2′-deoxyuridine (BrdU) cell proliferation assay (Roche Diagnostics GmbH, Mannheim, Germany), as reported by Giofrè and co-workers [29]. Briefly, 4 × 10^3^ cells/well were plated in a 96-well plate for 24 h. Then, cells were treated as previously described for MTT assay. Afterwards, cells were incubated with BrdU labeling solution for another 24 h at 37 °C followed by fixation and incubation with anti-BrdU peroxidase conjugate for an additional 1.5 h at room temperature. Finally, after substrate reaction, color intensity was measured with iMark™ microplate reader (Bio-Rad Laboratories) at a wavelength of 450 nm with reference at 690 nm. 

The viability was determined as the percentage of viable cells in treated cultures compared to those in untreated ones.

### 2.6. Determination of Reactive Oxygen Species and Mitochondrial Membrane Potential

Reactive oxygen species (ROS) and mitochondrial membrane potential (∆ψ_m_) were evaluated by flow cytometry. HUVECs were plated on 6-well plates (8 × 10^4^ cells/well) and treated as described for cell viability assays. Each sample was harvested, centrifuged at 1200 rpm for 5 min, washed with PBS, and resuspended in a serum-free medium. Aliquots of cell suspension were loaded with 10 µM DCFH-DA for 30 min at 37 °C and used to evaluate the intracellular ROS [30]. The signals of the highly fluorescent oxidized derivative 2′-7′-dichlorofluorescein (DCF), formed in the presence of ROS, were collected with Novocyte 2000 cytofluorimeter (Agilent, Santa Clara, CA, USA). To assess the trans-membrane potential (Δψm), we stained the HUVECs cells with 10 μM rhodamine 123 (R123, Invitrogen Molecular Probes), a fluorescent probe that accumulates in the matrix of functional mitochondria, for 10 min at 37 °C [31]. Signals were collected cytofluorimetrically.

### 2.7. Cytofluorimetric Evaluation of 8-oxo-dG

Oxidative DNA damage was assessed as levels of 8-Oxo-2′-deoxyguanosine (8-oxodG) employing the FITC-labeled avidin probe, which is highly affine to 8-hydroxyguanine (8-OH-Gua), being structurally like biotin. In brief, after treatment, cells were permeabilized with methanol at −20 °C for 15 min and incubated with avidin-FITC conjugate (0.2 µM) at 37 °C for 1 h [25]. The fluorescence was recorded by cytofluorimeter.

### 2.8. Measurement of Superoxide Dismutase and Catalase Activities and Glutathione Levels 

HUVECs were seeded at a density of 8 × 10^4^ cells/well in 6-well plates and were treated as previously described. Then, superoxide dismutase (SOD) and catalase (CAT) activities, along with glutathione (GSH) content, were measured using commercial assay kits (AbCam, Cambridge, MA, USA) according to the manufacturer’s protocols. The absorbance was recorded by an iMark™ microplate reader (Bio-Rad Laboratories) at 450 nm for SOD, 570 nm for CAT, and 405 nm for GSH [25].

### 2.9. Determination of NO Accumulation in HUVECs Culture Supernatant

The nitric oxide (NO) production was evaluated by a colorimetric commercial kit (Sigma-Aldrich). Supernatants were collected and processed as described by Ferlazzo and colleagues [32]. Absorbance was recorded at a wavelength of 540 nm by iMark™ microplate reader (Bio-Rad Laboratories).

### 2.10. Detection of Apoptotic Cell Death and Caspase-3 Enzymatic Activity 

The protective effect of RGJe against BPA-induced cell death was evaluated cytofluorimetrically by the Annexin V-fluorescein isothiocyanate (FITC)/propidium iodide (PI) staining [33,34]. The treated HUVECs were collected and processed as reported above. Finally, samples were run on a cytofluorimeter. Caspase 3 enzymatic activity was assessed using a commercial kit (AbCam) according to the manufacturer’s instructions. Absorbance was detected at 405 nm by iMark™ microplate reader (Bio-Rad Laboratories) [32].

### 2.11. Real Time-PCR 

The HUVECs were treated as previously described. Then, total RNA of treated or untreated cells was extracted and reverse transcribed following the previously described procedures [35,36]. Messenger RNA levels were analyzed by real-time PCR, using SYBR green as a fluorescent probe. The analysis was carried out on a 7300 Real-Time PCR System (Applied Biosystems, Waltham, MA, USA). The β-actin was used as housekeeping control, and a standard dissociation stage was included to assess primer specificity. Data were collected and analyzed using the 2^−∆∆CT^ relative quantification method. Values are presented as fold change relative to untreated cells. The primer sequences used for real-time PCR are listed in Table 1.

### 2.12. Statistical Analysis

One-way analysis of variance (ANOVA) was used to interpret the data. Multiple comparisons of the means of the groups were performed using Dunnett’s multiple comparison test (GraphPad Prism 8 Software, San Diego, CA, USA). *p*-values less than or equal to 0.05 were considered significant.

## 3. Results

### 3.1. Polyphenolic Profile of RGJe

Based on the available reference standards, a total of 20 polyphenols from diverse chemical classes, including phenolic acids, hydroxycinnamates, flavonols, flavanols, resveratrols, dihydroflavonols, dihydrochalcones, and flavones, were reliably determined in RGJe. The most abundant polyphenols were the flavonols kaempferol-3-glucoronide (1241.64 mg/kg) and quercetin-3-glucoronide (8341.89 mg/kg) and the cis isomer of resveratrol (3821.14 mg/kg). Other compounds determined in relevant amounts were the ellagic acid (1124.32 mg/kg) and the caffeic acid (1400.65 mg/kg). On the other hand, the least concentrated polyphenols were certain flavonols, such as rutin (14.90 mg/kg) and luteolin (39.49 mg/kg), as well as phenolic acids, such as methyl-gallate (14.55 mg/kg) and p-hydroxybenzoic acid (29.74 mg/kg). The quantitative determination of polyphenols identified in RGJe is listed in Table 2.

### 3.2. Antioxidant Activity of RGJe in Cell-Free Models

The antioxidant and radical scavenging properties of RGJe were proved by a range of tests, as described in the Materials and Methods. The total phenolic content, expressed as milligrams of gallic acid equivalents (GAE) per gram of RGJe and evaluated by the Folin–Ciocalteu method, was 80.0634 ± 1.4295. The valuable antiradical activity of RGJe, shown in the DPPH test and expressed as milligrams of Trolox equivalents (TE) per gram of extract (mg TE/g), was 69.7117 ± 9.3961, while in the Reducing Power test, it was 82.5972 ± 4.3769, expressed as milligrams of ascorbic acid equivalent (AAE) per gram of RGJe (mg AAE/g) (Table 3). Moreover, the obtained values of 130.7420 ± 9.2355 mol TE/g of RGJe demonstrated a high antioxidant capacity of RGJe against peroxyl radicals (Table 3).

### 3.3. RGJe Prevents BPA-Induced HUVECs Cell Death and Counteracts the Reduction of Cell Proliferation 

**Figure 1:**
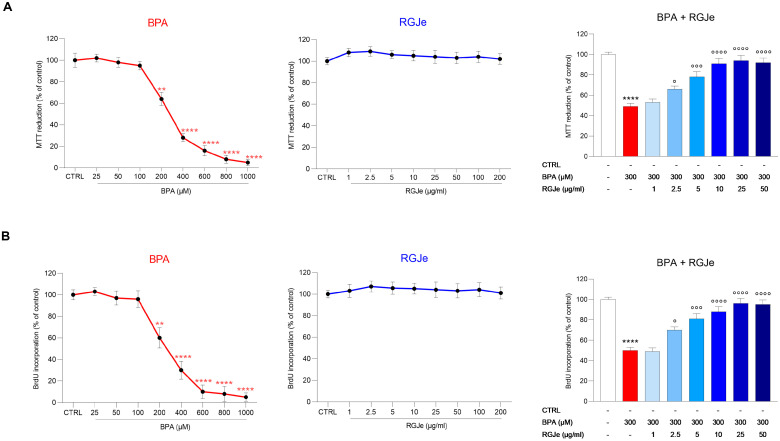
Protective effect of RGJe against BPA-induced cytotoxicity in HUVECs. Cell viability was evaluated by MTT test (**A**) and cell proliferation via BrdU incorporation test (**B**). Results of both assays are expressed as percentages ± SEM of the absorbance value detected in treated cells compared to untreated ones (control, CTRL). Three independent experiments in eight replicates were performed (n = 24). ** *p* < 0.01 and **** *p* < 0.0001 vs. CTRL; ° *p* < 0.05, °°° *p* < 0.001, and °°°° *p* < 0.0001 vs. BPA 300 µM.

Firstly, we evaluated the effect of BPA and RGJe alone on HUVECs’ viability for 24 and 48 h, respectively. The IC50 value for BPA was 303.91 μM; therefore, for further experiments, we decided to use BPA at a concentration of 300 μM (Figure 1A, left). Moreover, we observed that RGJe at all the tested concentrations (1–200 μg/mL) did not affect HUVECs’ cell death (Figure 1A, center). Therefore, we evaluated the potential protective effect of RGJe against BPA-induced cell death. The incubation of HUVECs with BPA (300 μM) reduced cell viability by 51 ± 3.05% compared to that of control cells (*p* < 0.0001; Figure 1A, right), while pre-treatment with RGJe at concentrations of 2.5, 5, 10, 25, and 50 μg/mL restored cell viability up to 66 ± 3.07%, 78 ± 5.06%, 91 ± 4.85%, 94 ± 5.12%, and 92 ± 4.53%, respectively (*p* < 0.05, *p* < 0.001 and *p* < 0.0001 vs. BPA; Figure 1A, right). On the contrary, pre-treatment of HUVECs with RGJe 1 μg/mL did not improve cell viability (Figure 1A, right). Data from the BrdU assay followed the same pattern as those of the MTT test (Figure 1B).

### 3.4. RGJe Counteracts BPA-Induced ROS Production and Restore the Loss of ΔΨm in HUVECs

**Figure 2:**
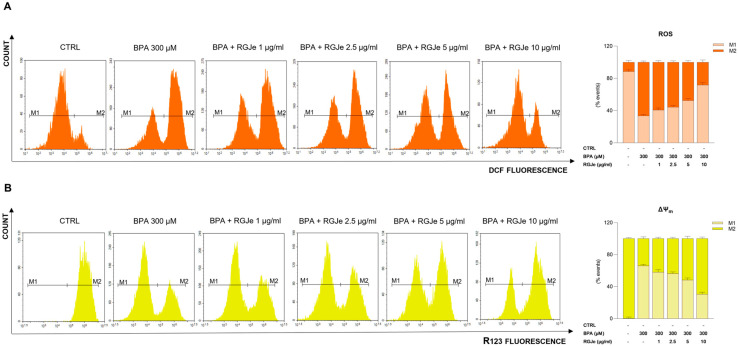
RGJe reduced both ROS generation and fall of ∆Ψm induced by BPA in HUVECs. Cytofluorimetric evaluation of intracellular ROS (**A**) and mitochondrial membrane potential (**B**) was carried out employing DCFH-DA and R123 fluorescent probes, respectively. Representative plots of three independent experiments performed in triplicate (n = 9) are shown. For ROS detection (**A**) the histograms show the percentage ± SEM of healthy cells (DCF−, M1) and cells with increased intracellular ROS (DCF^+^, M2) of three separate experiments in triplicate (n = 9). For ∆Ψm evaluation the histograms represent the percentage ± SEM of healthy cells (R123^+^, M2) and the cells with impaired mitochondrial membrane potential (R123−, M1) of three separate experiments in triplicate (n = 9).

The incubation of HUVECs with BPA (300 μM) caused a remarkable growth of intracellular ROS compared to untreated cells (Figure 2A). In details, we observed that 66.55 ± 1.5% of cells showed enhanced intracellular ROS after BPA exposure. Contrariwise, the pre-treatment with RGJe at 2.5, 5 and 10 µg/mL counteracted this effect (Figure 2A); indeed, the percentage of fluorescent cells were 55.73 ± 1.7%, 47.41 ± 2.1% and 28.14 ± 2.88%, respectively (Figure 2A). RGJe at concentration of 1 µg/mL did not evoke any significant reduction of ROS generation by BPA (Figure 2A).

Moreover, the exposure of HUVECs to BPA 300 μM for 24 h significantly affected the ΔΨm (Figure 2B). In particular, we observed that 33.93 ± 2.2% of cells are R123^+^ compared to untreated cells (99.79 ± 1.3%), showing a significant fall of ΔΨm. This effect was prevented by RGJe at 2.5, 5 and 10 µg/mL (43.64 ± 1.7%, 51.63 ± 2.9% and 69.42 ± 2% of R123^+^ cells, respectively). Also, the lowest concentration of RGJe (1 µg/mL) used restored, even with less extent, the loss of ΔΨm evoked by BPA (Figure 2B).

### 3.5. RGJe Protective Effects on BPA-Induced DNA Damage in HUVECs

**Figure 3:**
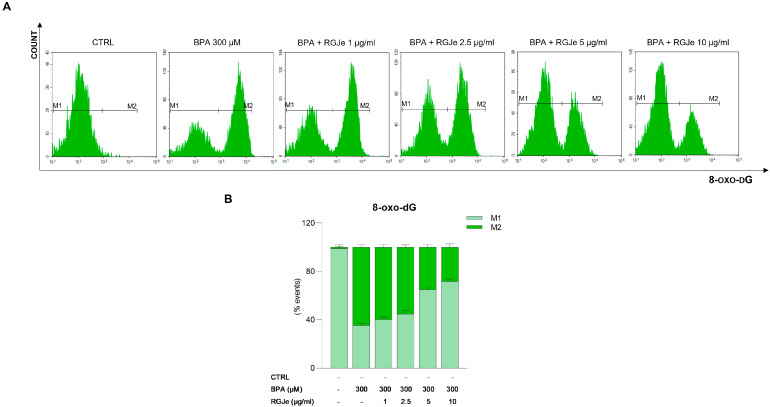
Protective effect of RGJe against the DNA oxidative damage induced by BPA in HUVECs. Levels of 8-oxo-dG were measured by flow cytometry, detecting the emission signals of fluorochrome FITC-labeled avidin. Representative plots of three different experimental sessions performed in triplicate (n = 9) are displayed (**A**). The histogram reports the percentage ± SEM of healthy (non-fluorescent, M1) and damaged (fluorescent, M2) cells (**B**), from three independent experiments performed in triplicate (n = 9).

To study the effectiveness of RGJe in attenuating DNA oxidative damage, we detected the levels of 8-oxo-dG by a FITC-conjugated avidin probe. As shown in Figure 3, the exposure of HUVECs to BPA 300 μM for 24 h induced DNA oxidation of 64.61 ± 2.3% compared to untreated cells. The pre-treatment with 5 and 10 μg/mL of RGJe for 24 h before the exposure to BPA decreased oxidative DNA damage by about 29.5 ± 2% and 36.4 ± 2.8%, relative to BPA alone-treated cells, respectively (Figure 3). The lowest concentrations of RGJe employed (1 and 2.5 µg/mL) restored, even with less extent, DNA damage elicited by BPA (Figure 3). The treatment with RGJe alone did not cause DNA oxidation, since the emission values almost overlapped those detected in untreated cells.

### 3.6. Effect of RGJe on Biomarkers of BPA-Induced Oxidative Stress in HUVECs

**Figure 4:**
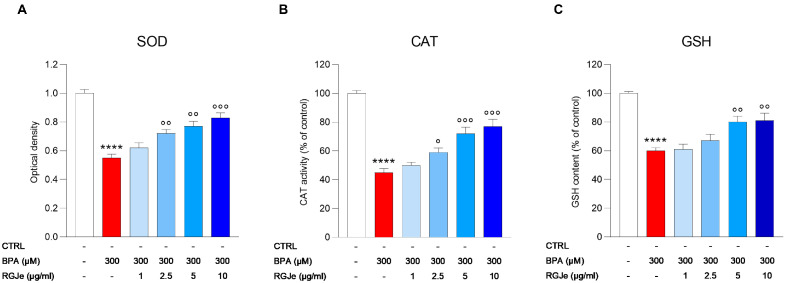
Effect of the pre-treatment with RGJe on antioxidant defense systems reduced by BPA in HUVECs. The activity of SOD (**A**) and CAT (**B**), as well as GSH levels (**C**) are reported. Data are expressed as the mean ± SEM of three separate experiments performed in triplicate (n = 9). **** *p* < 0.0001 vs. CTRL; ° *p* < 0.05, °° *p* < 0.01, °°° *p* < 0.001 vs. BPA 300 µM.

We measured the amount of SOD and CAT activities, as well as GSH content to determine the level of oxidative stress in HUVECs after BPA treatment with or without RGJe. As shown in Figure 4, the treatment of HUVECs with BPA caused a significant reduction of the levels of these biomarkers in comparison to untreated cells (45 ± 2.65%, 55 ± 2.8% and 40 ± 2.11% for SOD, CAT, and GSH, respectively, *p* < 0.0001). 

Pre-treatment with RGJe at concentration of 2.5 μg/mL (68 ± 2.75 and 59 ± 3 for SOD and CAT, respectively; *p* < 0.05), 5 μg/mL (76 ± 3.46 and 72 ± 4.4 for SOD and CAT, respectively; *p* < 0.01 and *p* < 0.001) and 10 μg/mL (83 ± 3.35 and 77 ± 5 for SOD and CAT, respectively; *p* < 0.001) for 24 h prior BPA exposure for further 24 h, significantly augmented the activity of both SOD and CAT (Figure 4A,B). The lowest concentration of RGJe tested in this study (62 ± 3.53 and 50 ± 2.13 for SOD and CAT, respectively) did not cause any significant improvement in these biomarkers (Figure 4A,B). The data obtained from the assessment of GSH content showed a similar trend even with lesser extent (Figure 4C). In fact, only the pre-treatment with RGJe at 5 and 10 μg/mL (80 ± 4.05 and 81 ± 5.11, respectively; *p* < 0.01) was able to significantly restore the GSH content affected by BPA (Figure 4C). Concentrations 1 and 2.5 μg/mL of RGJe did not exert any significant effect (Figure 4C). 

### 3.7. Effect of RGJe on NO Release Modulated by BPA in HUVECs 

**Figure 5:**
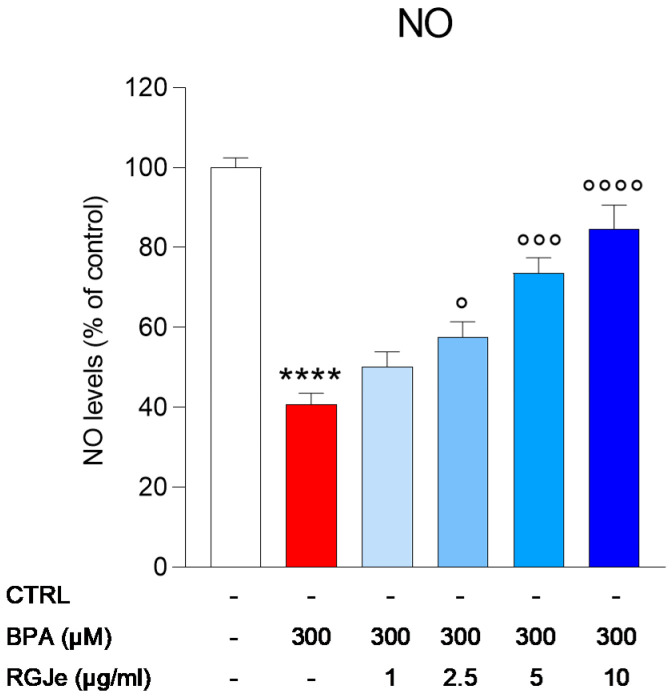
Effect of RGJe on reduction of NO release induced by BPA in HUVECs. The NO levels were determined by a colorimetric assay. Data are expressed as the percentage value of NO detected in treated cells compared to untreated ones and are shown as mean ± SEM from three independent experiments performed in triplicate (n = 9). **** *p* < 0.0001 vs. CTRL; ° *p* < 0.05, °°° *p* < 0.001, and °°°° *p* < 0.0001 vs. BPA 300 µM.

The incubation of HUVECs with BPA for 24 h caused about a 60 ± 2.76% reduction of NO production (*p* < 0.0001; Figure 5) that was significantly counteracted by pre-treatment with RGJe at 2.5, 5, and 10 μg/mL (42.5 ± 3.8%, 26.35 ± 3.7%, and 15.24 ± 5.86%, respectively; *p* < 0.05, *p* < 0.001, and *p* < 0.0001) compared to BPA-treated cells. Pre-treatment with the lowest concentration used in this study (1 μg/mL) did not induce any significant increase in NO levels. 

### 3.8. RGJe Reduces the Apoptotic Cell Death, Modulates Both Caspase 3 Activity and Apoptotic-Related Genes 

**Figure 6:**
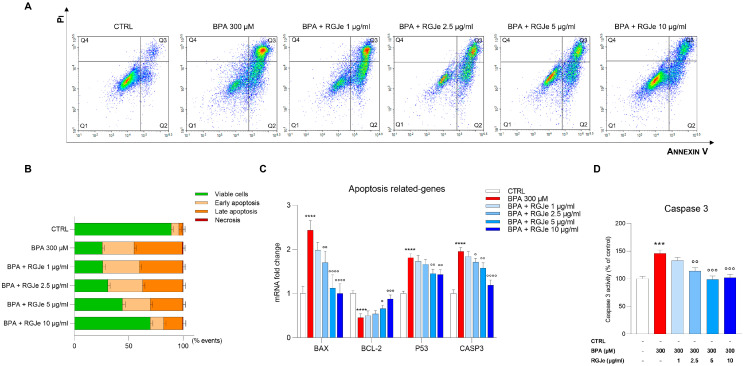
RGJe reduced the BPA-induced apoptotic cell death and modulated Casp3 enzymatic activity and apoptotic-related genes in HUVECs. Evaluation of apoptosis was performed cytofluorimetrically by the Annexin V-FITC/PI test (**A**). Representative Annexin V vs. PI dot plots are shown where necrotic (Annexin V−/PI+), late apoptosis (Annexin V+/PI+), viable (Annexin V−/PI−), and early apoptosis (Annexin V+/PI−) cells are in Q4, Q3, Q1, and Q2, respectively. The histograms report the percentage of cells for each quadrant, expressed as the mean ± SEM of three experiments in triplicate (n = 9) (**B**). The mRNA levels of apoptosis-related genes BAX, BCL-2, P53, and CASP3 were quantified via RT-PCR using the 2^−ΔΔCT^ method. Data are expressed as n-fold change relative to the untreated cells, reporting the obtained values from three independent experiments performed in triplicate (n = 9) (**C**). Data of caspase 3 enzymatic activity are expressed as the percentage of the value detected in treated cells compared to the untreated ones and are displayed as the mean ± SEM of three experiments in triplicate (n = 9) (**D**). *** *p* < 0.001 and **** *p* < 0.0001 vs. CTRL; ° *p* < 0.05, °° *p* < 0.01, °°° *p* < 0.001, and °°°° *p* < 0.0001 vs. BPA 300 µM.

The protective effect induced by RGJe was also evaluated cytofluorimetrically by Annexin V-FITC/PI assay. As shown in Figure 6A,B, the incubation of HUVECs with BPA for 24 h increased the percentage of cells in early (22.26 ± 1.9%, Annexin V+/PI−) and late (40.69 ± 2.1%, Annexin V+/PI+) apoptosis compared to untreated cells (Figure 6A,B). The pre-treatment with RGJe for 24 h prior to the incubation with BPA 300 μM reduced the number of cells undergoing apoptosis. In particular, the RGJe 5 and 10 μg/mL decreased the percentage of early apoptosis by 3.58 ± 1.9% and 16.82 ± 2%, respectively, while that of late apoptosis was reduced to 14.4 ± 2.2% for 5 μg/mL and 27.03 ± 2.7 for 10 μg/mL, in comparison to BPA-treated cells (Figure 6A,B). The two lowest concentrations of RGJe tested in this study only partially reduced BPA-induced apoptosis in HUVECs (Figure 6A,B). 

These results were supported by the evaluation of caspase 3 activity assay. The exposure to BPA for 24 h increased caspase 3 activity of HUVECs compared to that of untreated ones by 45.86 ± 6% (*p* < 0.001; Figure 6D). Pre-treatment with RGJe (2.5, 5, or 10 μg/mL) for 24 h reduced the activity of caspase 3 induced by BPA of 32.06 ± 6.08%, 46.86 ± 6.09%, and 43.99 ± 5.97%, respectively (*p* < 0.01 and *p* < 0.001), while RGJe at 1 μg/mL concentrations had no significant effect on this enzymatic activity (Figure 6D). A similar fashion was found by examining the mRNA levels of CASP3 by RT-PCR (Figure 6C).

Moreover, as shown in Figure 6C, the incubation of HUVECs with BPA for 24 h significantly enhanced the mRNA levels of the pro-apoptotic BAX and P53 genes up to 2.43 ± 0.12 and 1.81 ± 0.05-fold, respectively (*p* < 0.0001), together with decreasing those of the anti-apoptotic BCL-2 up to 2.21 ± 0.05-fold down (*p* < 0.01). These effects were significantly hampered by pre-exposure to RGJe at both 5 (2.17 ± 0.17-fold down, *p* < 0.0001, and 0.66 ± 0.04-fold, *p* < 0.05, for BAX and BCL-2, and 1.25 ± 0.06-fold down, *p* < 0.01, for P53, vs. BPA) and 10 μg/mL concentrations (2.44 ± 0.13-fold down, *p* < 0.0001, for BAX and 0.88 ± 0.05-fold, *p* < 0.001, and 1.26 ± 0.06-fold down, *p* < 0.01, for BCL-2 and P53, vs. BPA) (Figure 6C). The mRNA levels of BAX were also modulated by pre-treatment with 2.5 μg/mL (1.43 ± 0.015-fold down, *p* < 0.01, vs. BPA) (Figure 6C).

### 3.9. RGJe Reduces the IL-8, IL-1β and MCP-1 mRNA Expression Induced by BPA in HUVECs

**Figure 7:**
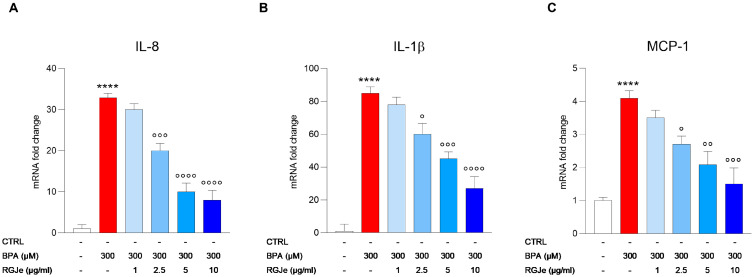
Effect of RGJe on IL-8 and IL-1β cytokines and MCP-1 chemokine gene expression in BPA-treated HUVECs. The results from RT-PCR of IL-8 (**A**), IL-1β (**B**), and MCP-1 (**C**) are expressed as an n-fold change compared to untreated cells after normalization against β-actin as endogenous control. Data represent the mean ± SEM of three different sets of experiments performed in triplicate (n = 9). **** *p* < 0.0001 vs. CTRL; ° *p* < 0.05, °° *p* < 0.01, °°° *p* < 0.001, and °°°° *p* < 0.0001 vs. BPA 300 µM.

As shown in Figure 7, BPA exposure (300 μM) of HUVECs for 24 h caused a massive increase of the mRNA levels of cytokines IL-8 (Figure 7A) and IL-1β (Figure 7B) in comparison to untreated cells. Pre-treatment of HUVECs with RGJe reduced the mRNA levels of these inflammatory mediators, with the highest effect at 5 and 10 μg/mL (Figure 7A,B). In particular, the IL-8 mRNA increase was 32.9 ± 1.04-fold (*p* < 0.0001) compared to untreated cells (Figure 7A). This increase was hampered by pre-treatment with RGJe at 2.5 (1.64 ± 1.80-fold down, *p* < 0.001), 5 (3.29 ± 2.13-fold down, *p* < 0.0001), and 10 (4.11 ± 2.34-fold down, *p* < 0.0001) μg/mL (Figure 7A). The IL-1β rise was 85 ± 3.8-fold (*p* < 0.0001) in comparison to control cells (Figure 7B). This effect was counteracted by the exposure to RGJe at 2.5 (1.42 ± 6.5-fold down, *p* < 0.05), 5 (1.9 ± 4.2-fold down, *p* < 0.001), and 10 (3.15 ± 7.2-fold down, *p* < 0.0001) μg/mL for 24 h before the incubation with BPA (Figure 7B).

In parallel, we detected the mRNA levels of MCP-1, a key chemokine that regulates the migration and infiltration of monocytes/macrophages. As shown in Figure 7C, BPA treatment of HUVECs for 24 h evoked an increase of 4.1 ± 0.22-fold (*p* < 0.0001) of mRNA expression of MCP-1 that was prevented by exposure of cells to RGJe at 2.5 (1.52 ± 0.25-fold down, *p* < 0.05), 5 (1.96 ± 0.39-fold down, *p* < 0.01), and 10 (2.73 ± 0.48-fold down, *p* < 0.001) μg/mL for 24 h prior BPA treatment (Figure 7C). RGJe at a concentration of 1 μg/mL did not induce any significative effect in counteracting the BPA-induced increase of IL-8, IL-1β, and MCP-1 (Figure 7).

### 3.10. RGJe Hampers the mRNA Increase of VCAM-1, ICAM-1 and E-Selectin Adhesion Molecules Induced by BPA in HUVECs

**Figure 8:**
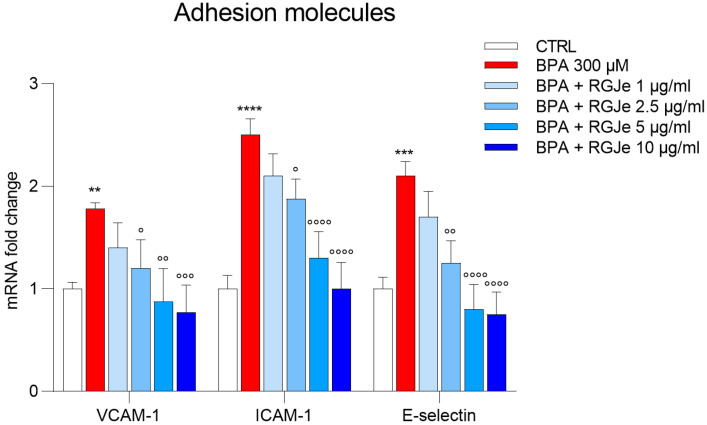
RGJe reduced the BPA-induced mRNA increase of VCAM-1, ICAM-1, and E-selectin adhesion molecules in HUVECs. The results from RT-PCR are expressed as an n-fold change compared to untreated cells, after normalization against β-actin as endogenous control. Data represent the mean ± SEM of three different sets of experiments performed in triplicate (n = 9). ** *p* < 0.01, *** *p* < 0.001, and **** *p* < 0.0001 vs. CTRL; ° *p* < 0.05, °° *p* < 0.01, °°° *p* < 0.001, and °°°° *p* < 0.0001 vs. BPA 300 µM.

As shown in Figure 8, the treatment of HUVECs with BPA 300 μM for 24 h determined a great increase of the mRNA levels of VCAM-1 (1.78 ± 0.03-fold; *p* < 0.01), ICAM-1 (2.5 ± 0.09-fold; *p* < 0.0001), and E-selectin (2.1 ± 0.08-fold; *p* < 0.001) in comparison to those found in untreated cells (Figure 8). This effect was counteracted by pre-treatment with RGJe at 2.5 μg/mL (1.48 ± 0.16-fold down, *p* < 0.05, 1.33 ± 0.11-fold down, *p* < 0.05, and 1.68 ± 0.13-fold down, *p* < 0.01, for VCAM-1, ICAM-1, and E-selectin, respectively), 5 μg/mL (2.03 ± 0.19-fold down, *p* < 0.01, 1.92 ± 0.15-fold down, *p* < 0.0001, and 2.62 ± 0.14-fold down, *p* < 0.0001, for VCAM-1, ICAM-1, and E-selectin, respectively) and 10 μg/mL (2.31 ± 0.15-fold down, *p* < 0.001, 2.5 ± 0.15-fold down, *p* < 0.0001, and 2.8 ± 0.13-fold down, *p* < 0.0001, for VCAM-1, ICAM-1, and E-selectin, respectively) (Figure 8).

## 4. Discussion

Grapes are one of the most widely grown fruits worldwide, with Europe, followed by Asia and the Americas, leading the world’s producers index of the winemaking history of the various countries [37]. Apart from wine, grapes are acknowledged for the wide plethora of biological activities, such as anticancer [38], anti-obesity [39], and anti-microbial [40], as well as the most relevant one, namely, antioxidant [41]. These effects are due to the richness of bioactive compounds, mostly polyphenols, present in the various part of the grape (i.e., skin, pulp, and seeds). Indeed, grapes’ major polyphenols include flavan-3-ols, flavonols, and anthocyanins, whereas other non-flavonoids typically consist of phenolic acids and stilbenes. Proanthocyanidins, which account for the majority of flavan-3-ols in grapes, are a family of chemicals formed by the fusion of flavanol monomers [42]. Anthocyanins are hydrophilic pigments mainly concentrated in the outer part of colored grape cultivars (i.e., red or purple grapes) [43]. In the RGJe employed in our study, kaempferol-3-glucoronide, quercetin-3-glucoronide, and the cis-isomer of resveratrol were the most abundant flavonoids found, followed by ellagic and caffeic acids, whose composition trace those already published [44].

As stated, polyphenols of grapes are well-established natural antioxidants. Indeed, it has been proven that they have radical scavenging, ion chelating, and reducing activities [41]. In our study, we found that RGJe possesses noteworthy antioxidant effects, as demonstrated by several abiotic assays, thus reinforcing the value of our phytocomplex. This is because oxidative stress, defined as an imbalance in the body’s oxidation and antioxidation condition, plays a significant role in accelerating cellular degeneration, leading to a variety of illnesses [45]. Environmental contaminants, such as BPA, strongly impair the body’s defenses due to an overwhelming increase in oxidative stress. This leads to a loss of the physiological functionality of various organs, with the cardiovascular system being one of the most affected [5]. In our study, BPA impaired the viability of HUVECs, employed as an in vitro model of vascular architecture, whereas pre-treatment with RGJe was able to hamper the harm induced by the toxicant, following a concentration-dependent fashion. Our results are in line with other studies reporting the potentiality of both grape seed and green tea extracts, rich in polyphenols, to hinder the toxicity of BPA in both HUVECs and isolated rat aorta [46,47]. 

These protective effects have been claimed to be principally due to polyphenols’ antioxidant potential. Indeed, we observed that pre-treatment with RGJe was able to lower intracellular ROS, as well as restore mitochondrial functionality after exposure to BPA. Moreover, RGJe effectively counteracted BPA-induced DNA oxidative damage, as showed by the evaluation of oxidized nucleosides. Notably, grape juice has already displayed protective effects against oxidative damage towards mitochondria and nucleic acids in cadmium-induced redox stress in rats, another alarming environmental contaminant under the spotlight of scientific community [48], thus corroborating grape juice antigenotoxic potential [49]. Furthermore, we proved that RGJe was able to boost two of the most powerful antioxidant enzymes, namely SOD and CAT, which are responsible for the detoxification of intracellular ROS over-production and were both hampered by the exposure of BPA. Moreover, it caused a depletion of GSH content, due to HUVECs’ demand to counteract the witnessed abnormal oxidative stress, an effect reverted by the pre-treatment with RGJe. BPA also decreased NO levels, since it is known that vascular oxidative stress lowers NO availability, thus stimulating endothelial dysfunction [50], an event that was significantly reverted by RGJe. Proanthocyanidins of grape peels and seeds have been studied for these activities in other experimental models, showing their ability to counteract different pro-oxidant stimuli via the increase of antioxidant enzymes [51,52].

Endothelial dysfunction caused by the imbalance between ROS production and removal by nonenzymatic and enzymatic antioxidant systems is claimed to cause apoptosis and necrosis in endothelial cells, promote the accumulation of macrophage-derived cytokines, and increase endothelial permeability [53]. In our study, exposure to BPA unleashed apoptosis of HUVECs, the index of cellular distress. This outcome was reverted by RGJe pre-treatment, which also modulated the whole apoptotic machinery via the increase of anti-apoptotic genes, such as BCL-2, and lowered the pro-apoptotic ones, such as BAX, P53, and CASP3, as well as the latter’s enzymatic activity.

Regarding the effect on cytokine production induced by BPA exposure in HUVECs, we witnessed a massive release of IL-8, IL-1β, and MCP-1, whose over-expression induces cell migration and adhesion, angiogenesis, and vascular permeability exacerbating atherosclerosis. RGJe was able to block this inflammatory-driven process, lowering the gene expression of these three chemokines. Our results are in line with previous reports suggesting that an anthocyanin-rich extract of *Aronia meranocalpa* attenuated the expressions of inflammatory cytokines in HUVECs stimulated with TNF-α [54].

**Figure 9:**
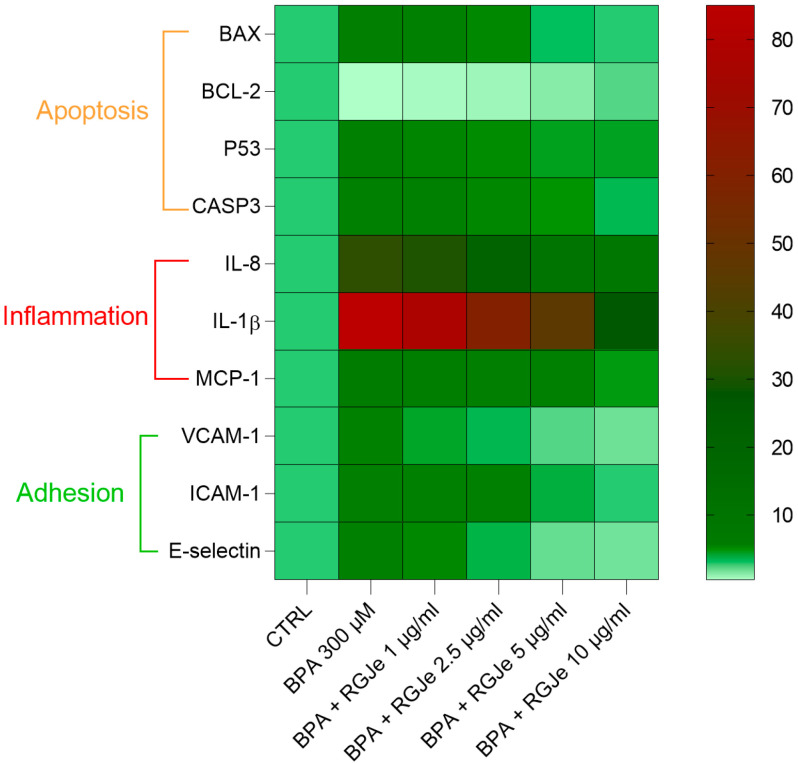
Heatmap of gene expression modulated by RGJe. The columns of the heatmap represent each treatment and the rows indicate the genes. Each cell is colored based on the amount of the gene found in that sample.

In human atherosclerotic lesions, endothelial cells have been shown to over-express intracellular VCAM-1, ICAM-1, and E-selectin in order to promote leukocyte adhesion and eventually stimulate endothelial dysfunction [55]. In this study, BPA expectedly enhanced the expression of the above-mentioned adhesion molecules, which is proof of its pro-inflammatory effect in HUVECs and its atherogenic activity. Notably, pre-treatment with RGJe thwarted this effect, restoring the physiologic levels of VCAM-1, ICAM-1, and E-selectin. In this field, others have discussed the protective effects of polyphenols from grapes, such as ellagic acid and anthocyanidins, in hampering the over-expression of adhesion molecules in endothelial cells after pro-inflammatory stimuli [56,57]. The heatmap of the genes regulated by RGJe in HUVECs exposed to BPA is shown in Figure 9.

## 5. Conclusions

Environmental pollutants represent an alarming issue for industrialized countries, given the resulting impairment of human health and the consequent socio-economic costs. Nutraceutical approaches may represent a hope for maintaining and defending the well-being of subjects exposed to these toxicants. In this study, we have shed light on the potentiality of RGJe as a tool for boosting one self’s antioxidant defense, severely hampered by environmental pollutants. This is particularly relevant for the cardiovascular system, whose damaged functionality, by BPA for example, may lead to atherosclerosis. In addition to the remarkable antioxidant properties, RGJe blocked apoptosis of endothelial cells as well as chemokine release and adhesion molecules increase induced by BPA. Our results sustain the value of RGJe in counteracting endothelial damage induced by environmental pollutants, laying the foundation for its possible employment in this field.

## Figures and Tables

**Table 1:** Oligonucleotide primers of genes used for real-time PCR.

Gene Product	NCBI Reference Sequence	Primer Sequence
BAX	NM_138764.5	Forward: 5′-GGACGAACTGGACAGTAACATGG-3′ Reverse: 5′-GCAAAGTAGAAAAGGGCGACAAC-3′
BCL-2	NM_000657.3	Forward: 5′-ATCGCCCTGTGGATGACTGAG-3′ Reverse: 5′-CAGCCAGGAGAAATCAAACAGAGG-3′
CASP3	NM_004346.4	Forward: 5′-AGCACCTGGTTATTATTCTTGG-3′ Reverse: 5′-GCTTGTCGGCATACTGTT-3′
IL-8 (CXCL8)	NM_000584.4	Forward: 5′-ACACTGCGCCAACACAGAAATTA-3′ Reverse: 5′-TTTGCTTGAAGTTTCACTGGCATC-3′
IL-1β	NM_000576.3	Forward: 5′-AGCCATGGCAGAAGTACCTG-3′ Reverse: 5′-TGAAGCCCTTGCTGTAGTGG-3′
MCP-1 (CCL2)	NM_002982.4	Forward: 5′-TCTCTTCCTCCACCACTATGCA-3′ Reverse: 5′-GGCTGAGACAGCACGTGGAT-3′
VCAM-1	NM_001078.4	Forward: 5′-ATGTCAATGTTGCCCCCAGA-3′ Reverse: 5′-ACAGGATTTTCGGAGCAGGA-3′
ICAM-1	NM_000584.4	Forward: 5′-AGCTTCGTGTCCTGTATGGC-3′ Reverse: 5′-CTGGCACATTGGAGTCTGCT-3′
E-selectin (SELE)	NM_000450.2	Forward: 5′-TCAAGTGTGAGCAAATTGTGAAC-3′ Reverse: 5′-ATTCTCCAGAGGACATACACTGC-3′
β-Actin	NM_001101.5	Forward: 5′-TTGTTACAGGAAGTCCCTTGCC-3′ Reverse: 5′-ATGCTATCACCTCCCCTGTGTG-3′

**Table 2:** Polyphenolic profile of RGJe revealed by RP-UHPLC-MS analysis. Results are expressed as mg/kg of dried extract and are reported as mean ± standard deviation of triplicate measurements. For each compound, the observed deprotonated molecular ion [M-H]^−^ and the retention time (t_R_) are also listed.

Chemical Class	Compound	[M-H]^−^ (*m*/*z*)	t_R_ (min)	Concentration (mg/kg)
Phenolic acids	2,6-diOH-benzoic acid	149.17	5.92	182.79 ± 15.02
	p-hydroxybenzoic acid	137.12	6.12	29.74 ± 2.87
	gallic acid	168.95	6.27	129.34 ± 11.23
	methyl-gallate	183.15	7.28	14.55 ± 1.40
	ellagic acid	301.19	16.10	1124.32 ± 123.56
	vanillic acid	167.14	13.80	338.34 ± 20.61
Hydroxycinnamates	caffeic acid	179.16	10.96	1400.65 ± 157.34
	p-coumaric acid	163.04	12.41	156.41 ± 12.05
Flavonols	luteolin	285.24	7.87	39.49 ± 3.69
	kaempferol-3-glucoronide	461.45	10.76	1241.64 ± 117.29
	quercetin-3-glucoronide	477.41	12.02	8341.89 ± 726.43
	rutin	609.25	12.80	14.90 ± 2.03
	quercetin	301.23	15.88	89.31 ± 7.58
Flavanols	catechin	289.27	9.58	673.69 ± 60.54
	epicatechin	289.26	11.26	420.14 ± 24.79
Dihydroflavonols	dihydrokaempferol	287.25	15.03	84.39 ± 10.21
Dihydrochalcones	phlorizin	435.42	13.30	57.42 ± 4.87
Flavones	luteolin-7-O-glucoside	447.37	10.45	74.38 ± 8.64
Resveratrols	trans-piceid	389.35	18.48	76.92 ± 9.03
	cis-piceid	389.36	18.55	3821.14 ± 355.41
Total polyphenols				18,311.45 ± 1134.79

**Table 3:** Antioxidant activity of RGJe evaluated by abiotic assays. Results are reported as mean ± SEM of three experiments performed in triplicate and expressed in standard equivalent/g of dried extract.

Folin-Ciocalteu (mg GAE */g)	80.0634 ± 1.4295
DPPH (mg TE **/g)	69.7117 ± 9.3961
Reducing Power (mg AAE ***/g)	82.5972 ± 4.3769
ORAC (mol TE **/g)	130.7420 ± 9.2355

* Gallic acid equivalents; ** Trolox equivalents; *** ascorbic acid equivalents. DPPH: 2,2-Diphenyl-1-picrylhydrazyl; ORAC: oxygen radical absorbance capacity.

## Data Availability

Not applicable.
